# Leading a Statistical Bioinformatics Lab: It's All About Finding Balance

**DOI:** 10.1371/journal.pcbi.1003333

**Published:** 2013-11-07

**Authors:** Olga Vitek

**Affiliations:** Department of Statistics, Department of Computer Science, Purdue University, West Lafayette, Indiana, United States of America; University of Bremen, Germany

## How Did Your Lab Start, and How Many People Are in the Lab?

**Start of the lab:** 2006

**Size of the lab:** Six PhD students

**Research field:** Statistical proteomics and bioinformatics

After obtaining an undergraduate degree from Switzerland, I became a PhD student in the Department of Statistics at Purdue University. From the first days in graduate school I became interested in interdisciplinary projects. My dissertation focused on a problem in structural biology, co-supervised by Prof. Bruce Craig from the Department of Statistics and Prof. Chris Bailey-Kellogg from the Department of Computer Science. In 2005, I did a one-year post-doc in mass spectrometry-based proteomics at the Institute for Systems Biology, under the direction of Prof. Ruedi Aebersold. In 2006, I returned to Purdue as a faculty member in the Department of Statistics and the Department of Computer Science, and started my own statistical proteomics and bioinformatics lab. Now the lab has six members, all PhD students, as post-docs and staff scientists are less common in our department.[Fig pcbi-1003333-g001]

**Image 1 pcbi-1003333-g001:**
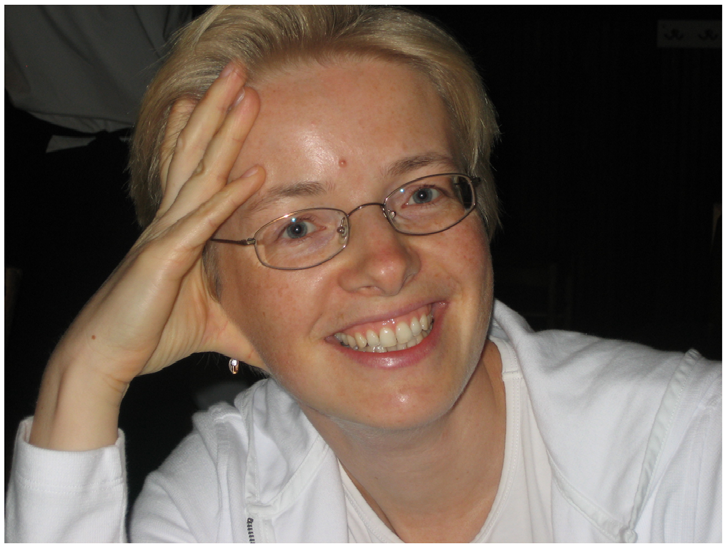
Olga Vitek.

## What Is the Scientific Mission of Your Lab?

Defining the scientific mission and the identity of a statistical bioinformatics lab can be difficult. Since statisticians do not conduct wet lab experiments, our role is often erroneously perceived as that of support. Fortunately, life sciences research is increasingly data driven. Statisticians have many opportunities to develop stand-alone methodological components of the life scientist's toolbox, which drive the experimentation and enhance reproducible research. New experimental technologies are also opportunities for developing novel statistical methods of general interest, e.g., handling large data sets, data visualization, scalable inference, and use of domain-specific information in model-based conclusions. At the same time, the diversity and the evolution of this area is such that both statisticians and experimentalists face steep learning curves.

I view the mission of my lab as a balancing act between consulting-like efforts, which are essential for understanding the biotechnological problems and the data, and method development efforts that lead to our main contributions. My approach to achieving this balance is to be an active member of both statistical and life science research communities, helping to close the communication gap between the two disciplines, and train interdisciplinary scientists.

## How Do You Hire Members of the Lab?

I have a detailed conversation with every prospective student regarding mutual expectations. My expectations are to assist with consulting for our collaborators, to carry out method development, to write software, and to author three publications (published manuscripts or extensive drafts) that serve as three main chapters of the dissertation. Most students have long-term interests and professional goals. I make an effort to understand their goals, and do my best to find a balance between these goals and the goals of the lab. This is good for the students, but it is also good for the lab, because this makes us all more productive. When a student joins the lab, I make a strong commitment to see him or her through until the completion of the degree. However, I expect a similarly strong commitment on the student's behalf to his or her projects.

While the technical skills are important, they are secondary at the hiring stage. The main factors are the motivation to pursue the degree, scientific integrity, and ability to go the distance. The technical skills can be learned, but these other factors have to come from within. A consequence of this choice is that new students often need training, and my group has a reputation of having longer than average graduation time. I believe that this is unavoidable in interdisciplinary research.

## How Do You Interact with Members of Your Lab?

Some incoming students expect that I will provide them with the technical details of the project, much as an instructor would do in a class. I have a different attitude, and believe that my main role is to help them shift from a “student” mentality to that of a professional and expert. This means I gradually give the student the decision power in his or her scientific activities. During the first year in the lab, I often pair new students with our life sciences collaborators, and ask them to be in charge of a consulting project and assisting with statistical tasks. This experience often becomes a great motivator, as the students see that their work is valued, and it teaches them to work independently and communicate effectively. This experience also helps the students identify open methodological problems, and we work together to finalize the details. After that, I try to walk a fine line between helping and stepping out of the way.

*“Give the student the decision power in his or her scientific activities.”*

My lab doesn't have standing group meetings. As the lab is relatively small and the students' projects may not overlap, I try to avoid asking lab members to attend more meetings that are not directly relevant to their work. Instead, I have weekly individual meetings with every student, where he or she presents reports, and I provide a more direct and personalized feedback. I believe that this is useful for both sides. When necessary, we also have meetings with a subset of the lab members who work on related projects. At the same time, I encourage the students to collaborate, split tasks, and share expertise. I am happy to hear that some mini-group meetings happen without my involvement.

When difficult situations arise, I remind myself to always put science and people first. This requires to clearly decide which compromises are acceptable, and which ones are not. I never compromise upon quality of the science. Having established that, I try to do the right thing for the students. For example, during the last six years we celebrated the birth of six babies to students in the lab. We had to take into account their family circumstances and enable some flexibility in schedule and work. Certain projects may take longer, other projects may need more participants, or some opportunities may be missed. But I do not look for success at all costs.

## What about the Funding?

Obtaining and managing research funds was one aspect of the academic work for which I was least prepared. In my first years as a faculty member, I joined all the tutorials and workshops that I could find. It was helpful to read successful applications of senior colleagues, and to participate in NSF (National Science Foundation) and NIH (National Institutes of Health) grant review panels. Small start-up grants from Purdue University and Indiana University, data analysis projects for collaborators, consulting, and small industry gift funds were my lifeline before the first federal grant, and they allowed me to fund one or two students. A better understanding of the mindset of the funding agencies helped to better structure my applications.

I prefer spending funds sooner rather than waiting for a “perfect” opportunity. I also encourage students to apply for their own funds, be it small travel grants or long-term fellowships. Many conferences offer student volunteering opportunities in exchange of reduced fees, and this is an excellent opportunity for them to save money, and to network.

*“I prefer spending funds sooner rather than waiting for a ‘perfect’ opportunity.”*

## How Do You Manage Your Time?

I have always been interested in time management and productivity strategies and software. The “Getting Things Done” system by David Allen (and the related OmniFocus software) work well for me. One important decision was a personal “not to do” list of tasks that I decided not to do, once and for all. The list is informal, and I only keep it in my mind, however, it simplified a lot of my decisions and saved hours of doubt and guilt.

*“One important decision was a personal ‘not to do’ list of tasks.”*

I use other well-known time management techniques, e.g., schedule meetings back-to-back and reserve large blocks of research time, as if it was another meeting. I have a closed door policy, but can have short-notice appointments. I use Skype to save the commute time, and change physical locations often to stay focused. I also do a lot of yoga, which helps to put things in perspective.

## How Do You Choose and Maintain Collaborations?

Our best collaborators are unlike us. They think differently and have complementary expertise. This is natural, because the interactions are mutually beneficial, and the respective contributions are clear. This is also challenging, because it requires stepping out of our comfort zones. For example, many life scientists don't have a clear view research in statistics, and how to credit our efforts. Statisticians may under-appreciate the difficulties of wet lab work. A strong commitment and a clear statement of everyone's roles and expectations are key. The collaborations also need to work on a personal level. They can only result from mutual trust and respect, and a shared understanding of what it means to do quality research. I am fortunate to have collaborators who are great friends and colleagues, and joint projects are a lot of fun.

When a project starts, the single most important task is to communicate with our collaborators. This means to never assume anything about the experiment or the data, implement basic checks, and summarize the analysis and assumptions in writing. Lab members also seek feedback from the broader community of potential users, e.g., by presenting case studies in short courses and workshops.

*“Never assume anything about the experiment or the data.”*

## Are There Challenges for Women in Science?

It is true that some actions are perceived differently when they come from a woman. I chose to worry about these challenges only when I can do something about them. Academic PIs are uniquely positioned to make a positive change by creating opportunities for female students to lead projects, making independent decisions, and mentoring junior students. Sometimes just saying, “you can do it” is enough. I try to create these opportunities within my lab, and hope that the experience provides not only scientific training, but also the ability to navigate the world.

On a personal level, as a woman in science, worrying about gender issues is on my “not to do” list. Worries have a way of amplifying problems and holding me back. Focusing on the science is liberating.

## Do You Have Any Advice to a Beginning Researcher?

My advice is to talk to other scientists as much as you can. Establish a support network of colleagues who are at a similar career stage. They will be the most receptive and will provide the most pertinent advice. Talk to the department head and senior colleagues, as they can only appreciate your work if they know about it. Find senior mentors who have career paths that inspires you. But most importantly, do not get upset by setbacks, learn from mistakes, improve, and carry on.

*“Talk to other scientists as much as you can.”*

